# Functional Shoulder Outcome and Quality of Life Following Modified Muscle-Sparing Pectoralis Major Flap Surgery

**DOI:** 10.3390/healthcare9091158

**Published:** 2021-09-03

**Authors:** Tina Rauchenwald, Dominik Knierzinger, Daniel Dejaco, Clemens Hengg, Volker H. Schartinger, Gerhard Pierer, Herbert Riechelmann, Dolores Wolfram

**Affiliations:** 1 Department of Plastic, Reconstructive and Aesthetic Surgery, Medical University of Innsbruck, 6020 Innsbruck, Austria; tina.rauchenwald@tirol-kliniken.at (T.R.); gerhard.pierer@tirol-kliniken.at (G.P.); 2 Department of Orthopedics and Traumatology, Medical University of Innsbruck, 6020 Innsbruck, Austria; dominik.knierzinger@tirol-kliniken.at (D.K.); clemens.hengg@tirol-kliniken.at (C.H.); 3 Department of Otorhinolaryngology, Head and Neck Surgery, Medical University of Innsbruck, 6020 Innsbruck, Austria; daniel.dejaco@tirol-kliniken.at (D.D.); volker.schartinger@tirol-kliniken.at (V.H.S.); herbert.riechelmann@tirol-kliniken.at (H.R.)

**Keywords:** salvage laryngectomy, pharyngocutaneous fistula, pectoralis major muscle flap, shoulder function, quality of life, Constant Murley Score

## Abstract

Background: The pedicled pectoralis major muscle flap (PMMF) is a well established flap for fistula prophylaxis after salvage laryngectomy. To reduce donor site morbidity, we established a modified muscle-sparing harvesting technique. We herein investigate postoperative shoulder function and health-related quality of life (HRQOL). Methods: A chart review of patients receiving the modified muscle-sparing pectoralis major muscle flap between 2013–2020 was performed. Nineteen patients (male = 18, female = 1) were potentially eligible and six male patients were ultimately enrolled. Postoperative shoulder function was assessed on both sides (flap side versus non-flap side) using the Constant Murley Score and the Bak criteria. Health-related quality of life was assessed with the European Organization for Research and Treatment of Cancer Quality of Life Questionnaire in cancer patients (EORTC QLQ-C30) and head and neck cancer patients (EORTC H&N35). Results: No Constant Murley Score subscale was statistically significant (*p* ≥ 0.180). Bak criteria was overall rated “Good“. Solely upper extremity adduction force was significantly altered on the flap side (*p* = 0.039). Median EORTC QLQ-C30 score was 82.2 (IQR 11.1) on the functional scale and 10.3 (IQR 2.6) on the symptomatic scale. Median quality of life score was 75.0 (IQR 33.3) and median EORTC QLQ-H&N35 was 20.6 (IQR 9.8). Conclusions: Postoperative shoulder function after modified muscle-sparing pectoralis major muscle flap surgery is comparable to function of the healthy side with a significant deficiency in adduction force not compromising daily life in this small study cohort.

## 1. Introduction

Head and neck cancer (HNC) patients with advanced stages of squamous cell carcinoma (SCC) of the larynx or hypopharynx are often subject to radio-chemotherapy as first-line treatment for organ preservation (i.e., stage 3 disease) or sole curative first-line treatment due to inoperability (i.e., stage 4a or 4b) [1]. In 20%–25% of cases, incomplete response to first-line treatment is a recognized issue [2,3]. Further irradiation due to overlapping radiation volumes or chemotherapy alone are not considered curative treatment options in such cases. Second-line surgery, referred to as salvage laryngectomy (SLE), is the treatment of choice if persistent or recurrent HNC is considered resectable [4]. However, SLE is accompanied by a high risk of morbidity. Pharyngocutaneous fistulas are a common complication with an incidence of 30.9% [4,5]. Additional reconstructive surgery to transfer vital tissue to a previously irradiated surgical area is therefore often required [5]. In reconstructive head and neck surgery, the pedicled pectoralis major muscle flap (PMMF) is a well established option for fistula prophylaxis after SLE [6,7,8]. The PMMF offers multiple advantages over microsurgery such as simplicity of harvest and a low rate of postoperative complications [9]. Limitations of the PMMF include donor site morbidity in terms of aesthetic and functional outcome, as the conventional harvesting technique includes detachment of the entire muscle and leaves the patient with extensive scarring across the chest. Studies suggest impaired shoulder mobility after harvesting the PMMF as the pectoralis major muscle is the main muscle of the anterior chest wall [10,11,12,13]. We previously described a modified muscle-sparing harvesting method preserving the clavicular and upper sternocostal part of the pectoralis major muscle that is routinely applied at our hospital [14]. Muscle sparing is possible by raising the flap only supplied by its dominant pedicle, the pectoral branch of the thoracoacromial artery, which only enters the muscle at the sternocostal part [12,13]. We aim to reduce donor site morbidity in order to accommodate reintegration into daily life and to consequently improve quality of life (QoL).

To our knowledge, no study has yet investigated postoperative shoulder function in combination with health-related quality of life (HRQOL) questionnaires employing validated methods for each assessment.

The aim of this study was to evaluate postoperative shoulder function and health-related quality of life by applying a standardized assessment in patients with laryngeal or hypopharyngeal cancer undergoing SLE followed by reconstruction with our modified muscle-sparing PMMF. 

## 2. Materials and Methods

### 2.1. Patient Population

Between 2013 and 2020 a total of 19 HNC patients with persistent or recurrent laryngeal or hypopharyngeal SCC were treated at our hospital after first-line therapy and were potentially eligible. Inclusion criteria were previously performed fistula prophylaxis or fistula treatment after laryngectomy by reconstruction with the modified muscle-sparing PMMF [14], as confirmed by surgical reports obtained from patient charts. Excluded were deceased patients at time of recruitment, patients with HNC other than the larynx or the hypopharynx, patients with neuromuscular disease or muscle dystrophy, patients with health-related inability to participate in functional shoulder testing due to progressive disease and patients with a preoperatively existing severe shoulder pathology. Preexisting severe shoulder pathologies were excluded based on patients’ histories of shoulder injuries and dysfunctions as well as routinely performed contrast-enhanced staging computer tomography (CT) scans of the head and trunk.

**Figure 1:**
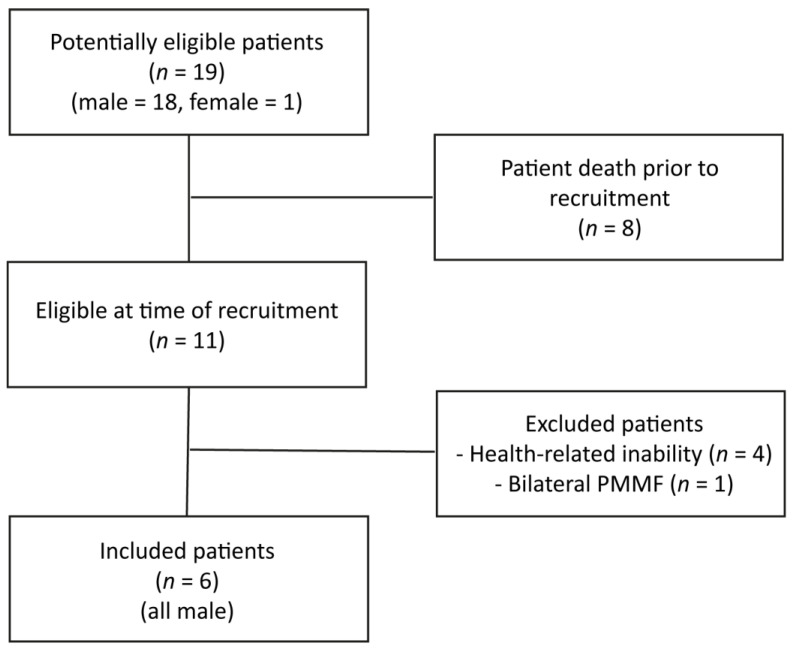
Patient inclusion process.

At the time of recruitment only eleven patients were still alive. Health-related inability to participate applied to four patients, who were in a palliative care setting due to progressive disease at the time of recruitment. One patient had to be excluded because a PMMF was applied bilaterally. The remaining six patients were enrolled in the present study and prospectively evaluated during routine oncologic follow-up visits (Figure 1). 

Written informed consent was obtained from all included patients in accordance with the Ethical Principles of the Declaration of Helsinki. Ethics approval was received from the Ethics Committee of the Medical University of Innsbruck (EK number: 1282/2019, EK vote: 20200209-2196). 

### 2.2. Functional Shoulder Assessment

Functional shoulder assessment was performed by the same orthopedic surgeon on both sides (operated “flap side” vs. non-operated “non-flap side”) in all patients. The examination included use of the Constant Murley Score (CMS), the Bak criteria and an ultrasound of both shoulders. During shoulder assessment, hand dominance was documented.

The CMS is an assessment tool originally established in 1987 to determine shoulder functionality after treatment of injury [15]. It combines two subjective and two objective subscales. The subjective subscales include “pain” rated on a Likert scale from 0 to 15 points and “activities of daily living” (ADL) rated on a Likert scale from 0 to 20 points. The objective subscales include “range of motion” (ROM) measured in degrees and “strength” measured in pounds (lbs.). These two scales are converted to score points from 0 to 40 for ROM and 0 to 25 for strength. Thus, the CMS results in a maximum total score of 100 points. Higher subscales as well as higher total scores indicate better shoulder function and less pain (Table 1).

The Bak criteria are an assessment tool used to evaluate the functional outcome following pectoralis major tendon repair in the case of pectoralis major tendon rupture described by Bak et al. [16]. These criteria combine three subjective and two objective parameters rated on an ordinal scale from “poor” to “excellent”. The subjective parameters include “pain”, “cosmetic complaints” and “activities”; the objective parameters include ROM and strength (Table 2).

Ultrasound examination of the shoulders was performed to exclude any secondary causes of decreased shoulder functionality in a standardized fashion by visualizing the long tendon of the biceps muscle and each tendon of the rotator cuff in two different planes to confirm its integrity or any partial or complete lesion. All examinations were performed using the same device (Esaote MyLabTM25Gold, Esaote SPA, Genua, Italy).

### 2.3. Health-Related Quality of Life Assessment

Assessment of HRQOL was performed as recommended by the European Organization for Research and Treatment of Cancer (EORTC). Patients completed the EORTC QLQ-C30 [17] and the EORTC H&N35 [18]. The EORTC QLQ-C30 is a validated questionnaire that is currently considered one of the most widely used cancer-specific HRQOL instruments in Europe. It consists of 30 questions building five functioning scales (physical, social, role, emotional, cognitive), nine symptom scales (fatigue, nausea and vomiting, pain, dyspnea, sleep disturbances, appetite loss, constipation, diarrhea, and financial impact), and a scale for global HRQOL. Scoring was done according to the EORTC QLQ-C30 scoring manual [19]. Raw scores were transformed to a scale from 0 to 100, with 100 reflecting the best possible score for functioning scales and the worst possible score for symptom scales.

The EORTC H&N35 is a validated HNC-specific subset that consists of 35 questions on one symptom (pain) and six functioning scales (swallowing, taste, smell, speech, social eating, sexuality). Again, scoring was done according to the EORTC H&N35 scoring manual [19] and scores were transformed to a scale from 0 to 100, with 100 reflecting the best possible score for functioning scales and the worst possible score for symptom scales.

### 2.4. Statistical Analysis

Patient clinical data were presented in tabular form. For continuous data, median and interquartile range (IQR) were calculated and compared with the Wilcoxon signed rank test, if applicable. For functional shoulder assessment the “non-flap side” served as reference. Data in the text are presented as “median (IQR)”. A significance level of 0.05 was defined. All calculations were performed with SPSS 26.0 (IBM Corp., Armonk, NQ, USA). 

## 3. Results

### 3.1. Patient Population

All included patients were male and median age at assessment was 63.5 (IQR 9.0) years. All included patients were diagnosed with SCC of the larynx. Five of the six patients underwent SLE followed by simultaneous pharyngocutaneous fistula prophylaxis using the muscle-sparing PMMF. One patient underwent reconstruction using the muscle-sparing PMMF for fistula treatment after previous laryngectomy. Three patients underwent radiation therapy and three patients radiochemotherapy prior to PMMF. Five of the six patients underwent bilateral neck dissection; one patient underwent neck dissection only on the PMMF side, compromising all patients equally on the PMMF side. All patients were right-handed. PMMF was taken from the left side in five cases and from the right side in one case to ensure coverage of a preexisting fistula on this side. Postoperative complications included hematoma in two patients. The first patient showed a hematoma caused by venous bleeding at the donor site and experienced partial flap loss during the postoperative course. Partial flap loss resulted in surgical debridement and coverage with an additional free flap. The second patient showed hematoma at the recipient site caused by bleeding from the muscle flap. This patient was further diagnosed with a pharyngocutaneous fistula, which was successfully treated by administering negative pressure therapy using EndoVAC. The clinical data are presented in Table 3.

### 3.2. Functional Shoulder Assessment

Median time between surgery and assessment was 24.5 (EQR 30) months.

**Figure 2:**
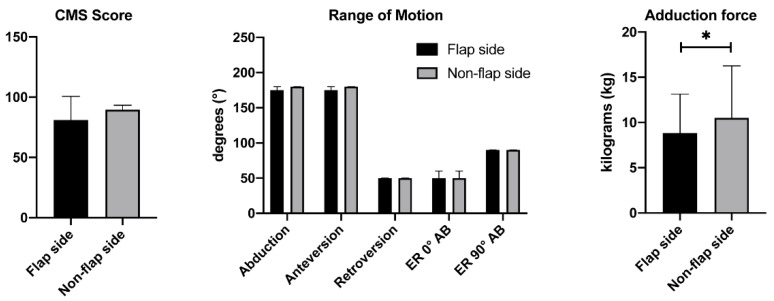
Constant Murley Score (CMS), median range of motion (ROM) in degrees (°) and median adduction force in kilograms (kg). Results are shown as median (IQR). A statistically significant difference was found in adduction force, *p* = 0.039 *.

Functional shoulder assessment including Constant Murley (CM) total score and CM subscale results are summarized in Table 4 and depicted in Figure 2. All differences between median CM score and subscale scores for the flap side vs. the non-flap side were not statistically significant (all *p* ≥ 0.180). One patient reported a mildly reduced ability to work and to do sports for the flap side in comparison to the non-flap side; another patient reported a moderately reduced ability to work and to do sports. None of the other patients reported any pain or restrictions. 

Differences in ROM between the flap side and the non-flap side were not statistically significant (all *p* ≥ 0.180) except for median adduction force. The difference in median adduction force was statistically significant with a *p*-value of *p* = 0.039.

Regarding internal rotation, patients were able to reach the gluteal to lumbal region with the arm of the flap side. In comparison, the arm of the non-flap side was able to reach the lumbal to thoracal region, performing a slightly better internal rotation on average.

**Figure 3:**
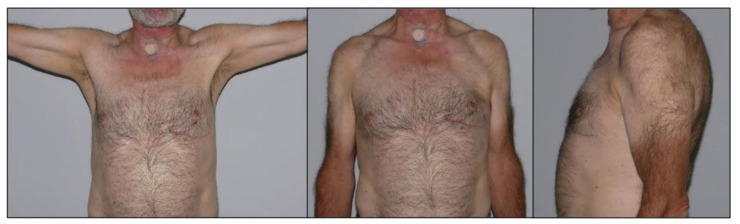
Clinical outcome in a patient after PMMF of the left side 135 days post surgery.

The Bak criteria regarding the flap side showed the result “Good“ in five patients and “Fair“ in one patient. None of the patients complained about cosmetic drawbacks and were pleased with aesthetic outcome (Figure 3). Clinically, slight volume deficits were observed on the flap side, while scars appeared flat with normal pliability and without contractions in all patients. The patient with the result “Fair” also showed the lowest CMS on both sides in comparison to the other patients. These results may be attributed to postoperative complications in this patient and a generally restricted state of health in regard to a reduced CMS on the healthy side.

Ultrasound examination of the shoulders revealed no major pathologies regarding the rotator cuff or the biceps tendon. In two patients, a partial tear of the supraspinatus tendon was observed on the non-flap side. As the patients were unaware of it and did not report any symptoms, the pathology was rated as non-severe and patients were able to participate in the functional shoulder assessment.

### 3.3. Health-Related Quality of Life Assessment

The median EORTC QLQ-C30 score was 82.2 (IQR 11.1) on the functional scale and 10.3 (IQR 2.6) on the symptomatic scale. Median quality of life (QoL) score was 75.0 (IQR 33.3). Altogether, only three out of a total of 180 questions were answered with the subjective feeling of strong restrictions, while the majority of all other answers were none or mild restrictions. One patient reported experiencing drawbacks in social activities with other people due to his medical condition. Another patient felt restrictions regarding his ability to work and pursue his hobbies. Median EORTC QLQ-H&N35 was 20.6 (IQR 9.8). All but one patient reported a strong reduction in their sense of smell and four of these patients reported experiencing severe difficulties talking on the phone attributed to prior laryngectomy.

## 4. Discussion

Despite modern microsurgical advancements, the PMMF remains a workhorse flap in the prevention of pharyngocutaneous fistula formation in patients undergoing SLE [20,21]. Significantly lower rates of postoperative morbidity have been observed in patients undergoing PMMF reconstruction as compared to patients undergoing free flap reconstruction [22]. Shortened operation time improves postoperative recovery and reduces further morbidity. Rehabilitation and reintegration into daily life are key as HRQOL is associated with overall survival in patients with HNC [23,24]. A higher HRQOL not only at diagnosis, but also post-treatment has been shown to result in improved survival rates [23]. Therefore, the choice of reconstructive method and the postoperative treatment may well be associated with oncological outcome. 

For evaluation, the EORTC QLQ-C30 has been shown to have a strong prognostic value in cancer patients [25]. Male HNC patients between 60 and 69 years of age show a mean EORTC QLQ-C30 score of 80.2 (±23.6) on the functional scale compared to a median score of 82.2 (IQR 11.1) in our patient collective and a mean score of 89.1 (±15.4) in age-matched, healthy male Austrian individuals [26,27]. We also found a higher QoL score with a median of 75.0 (IQR 33.3) in our patients in comparison to 64.9 (±23.6) shown as mean reference value in HNC patient [27]. Additionally, the mean QoL score of healthy male individuals was only slightly higher, namely 77.17 (±17.33) [26]. According to these findings, the symptom scale score was lower in our patients with a median score of 10.3 (IQR 2.6) compared to 15.7 (±24.8) on average in HNC patients, while it was comparable to a mean score of 9.9 (±16.4) in healthy male individuals [26,27]. These findings may be attributed to the fact that our patient collective included only patients in complete remission during survivorship compared to reference values for HNC patients at different disease stages. Only one of our patients showed a lower functional score (75.6) than the average, while their symptom score and QoL score were still better than the reference values. Scores of individual patients that vary by comparison to those of other patients or significant score changes in one patient should give rise to concern and follow-ups should possibly be intensified. 

One main aspect of QoL is reintegration into daily life. Functions such as adduction, forward flexion, and internal rotation of the humerus are attributed to the pectoralis major muscle [13]. Although the pectoralis major muscle is the main muscle of the anterior chest wall, it is not considered necessary for basic movements in everyday life [28]. However, previous work has reported impaired shoulder mobility after PMMF, without describing the technique of muscle harvest in detail [10,11,29,30]. This suggests that the conventional harvesting technique was applied, possibly resulting in larger muscle defects and thereby functional drawbacks. Xiao et al. compared postoperative outcome after oral cavity reconstruction using the PMMF or the anterolateral thigh perforator free flap. The Medical Outcomes Study-Short-Form-36 (MOS SF-36) and the University of Washington Quality of Life (UW-QOL) questionnaire were applied and patients reported better appearance and shoulder function after free flap reconstruction [10]. Hsing et al. also investigated postoperative outcome following oral cavity reconstruction using the PMMF in comparison to free tissue transfer. Using the UW-QOL only, patients after free flap reconstruction reported better shoulder function than did patients after PMMF [11]. Refos et al. noticed shoulder morbidity in terms of reduced ROM of abduction more frequently in patients with PMMF reconstruction and neck dissection than in patients with neck dissection only, suggesting that PMMF harvest adds to shoulder impairment [29]. Moukarbel et al. compared shoulder function of laryngectomized patients with neck dissection and PMMF employing the Shoulder Pain and Disability Index questionnaire, ROM and strength measurements. On the PMMF side, significantly reduced anteflexion and rotation were observed [30]. Other studies also observed reduced functional neck and shoulder outcome in HNC patients after neck dissection with greater impairment when PMMF was performed additionally [31,32,33]. Neck dissection alone, which was also performed in our patient collective, may already impair shoulder mobility by iatrogenic nerve injury or postoperative immobilization. As all our patients received bilateral neck dissection or neck dissection on the flap side, we may assume that all our patients are compromised comparably on the PMMF side. However, functional shoulder assessment after PMMF was not the main focus of the previously discussed studies.

In traumatology, a common mechanism of muscle rupture is avulsion of the pectoralis major tendon at its insertion on the proximal humerus or injury at the musculotendinous junction [34]. Our PMMF harvesting technique applied in these study patients specifically focuses on preserving functionality by sparing the clavicular and the superior sternocostal part of the pectoralis major muscle. In comparison to traumatologic injury and conventional harvesting techniques during reconstructive surgery, we resect only a muscle strip including the lower sternocostal and abdominal part, while the muscle tendon does not get detached, which lets us anticipate improved functional outcome [14]. According to Sun et al., a correlation exists between flap size and upper extremity dysfunction after PMMF with greater dysfunction in patients with larger flap size [35]. Postoperative functional shoulder assessment in our patients after modified muscle-sparing harvesting technique confirmed not only sufficient shoulder mobility by assessing ROM and CMS, but did not show a significant difference between the operated side and the healthy side with the exception of adduction force. Additionally, the non-flap side was the dominant side in five of our six patients. Furthermore, our patients show a better CMS with a median score of 89.5 (IQR 15.3) in comparison to patients presented by Merve et al. with a median CMS of 80 (range 48, 100) or 62 (range 49, 100) after modified radical neck dissection or radical neck dissection, both followed by reconstruction employing the conventional PMMF [36]. In fact, our patients showed a CMS comparable to that of healthy male individuals aged 61–70 years, who present a mean CMS of 90 (±2) according to Yian et al. [37]. The only significant difference detected in postoperative functional shoulder assessment in our patients was adduction force with reduced strength on the flap side. However, based on our QoL data, we may assume that an isolated reduced adduction force does not compromise the ability to participate in activities of daily life and, therefore, quality of life. 

Limitations of this study include the limited number of patients due to the high morbidity and mortality in this patient collective. Due to the nature of this study, we were solely able to evaluate the postoperative outcome in patients still alive, thus creating a selection bias over patients who deceased at an earlier date due to aggressive disease (*n* = 8) or who showed a health-related inability to participate (*n* = 4). Additionally, due to the known gender-related incidence of SCC of the larynx and hypopharynx, this study cohort happened to only represent male patients [38]. 

## 5. Conclusions

Functional shoulder outcome following the modified muscle-sparing harvesting technique of the PMMF is comparable with that of the healthy side in our patient collective as well as in healthy individuals of the same sex and age. In spite of reduced adduction force, overall postoperative shoulder function appears to not compromise participation in activities of daily life in this small cohort of oncologic high-risk patients.

## Figures and Tables

**Table 1:** Constant Murley Score (CMS) [15].

Constant Murley Score (CMS)	Points
Pain None Mild Moderate Severe	15 10 5 0
Activities of daily living (ADL) Activity level Full work Full recreation/sport Unaffected sleep Positioning Up to waist Up to xiphoid Up to neck Up to top of head Above head Total	4 4 2 2 4 6 8 10 20
Range of motion (ROM)	40
Strength (1 point per pound of weight)	25
Total	100

**Table 2:** Bak criteria [16].

Bak Criteria	Shoulder Function
Excellent	The patient was pain free, had a full range of motion, had no cosmetic complaints, had symmetrical manual adduction strength assessment or <10% isokinetic strength loss, and had returned to previous activities without restrictions.
Good	The patient had only slight functional impairment with only slight restrictions in movement or strength, and was without cosmetic complaints, with symmetrical manual adduction strength or <20% isokinetic deficit.
Fair	There was an impairment of function that affected return to desired activity; that is, pain or weakness on activity, or the cosmetic result was unsatisfactory.
Poor	Significant complications occurred, pain or restricted range of motion persisted, or there were significant cosmetic complaints from scarring or inadequate repair.

**Table 3:** Clinical characteristics.

No.	Sex	Age at Assessment	Tumor Site	Tumor Histology	TNM	Radiation/Radiochemo-Therapy Prior to PMMF	Indication for PMMF	Postoperative Complication	PMMF Side	Hand Dominance	Time between PMMF and Assessment (Months)	CMS (Flap/Non-flap Side)	BAK Criteria (Flap Side)
1	m	77	larynx	SCC	T1 N0 M0	R	prophylactic upon SLE	none	left	right	60	74/79	good
2	m	65	larynx	SCC	T2 N1 M0	R	tracheoesophageal fistula	hematoma, partial flap loss	right	right	32	95/93	good
3	m	54	larynx	SCC	T4a N0 M0	RC	prophylactic upon SLE	hematoma, fistula	left	right	17	44/72	fair
4	m	58	larynx	SCC	T3 N2b M0	RC	prophylactic upon SLE	none	left	right	42	89/92	good
5	m	62	larynx	SCC	T1b N0 M0	R	prophylactic upon SLE	none	left	right	4	94/94	good
6	m	69	larynx	SCC	T4a N0 M0	RC	prophylactic upon SLE	none	left	right	7	90/87	good

**Table 4:** Functional shoulder assessment results. A statistically significant difference was found in adduction force, *p* = 0.039 *.

Functional Shoulder Assessment	Flap Side Median (IQR)	Non-Flap Side Median (IQR)	*p*-Value
CMS	89.5 (15.3)	89.5 (11.8)	0.279
subscale “pain”	15.0 (0.0)	15.0 (0.0)	1.000
subscale “ADL”	18.0 (6.0)	19.0 (2.0)	0.180
subscale “ROM”	36.0 (1.5)	36.0 (3.0)	0.180
subscale “strength”	20.5 (5.3)	20.5 (7.0)	0.588
Abduction (°)	175.0 (10.0)	180.0 (7.5)	0.180
Anteversion (°)	175.0 (10.0)	180.0 (7.5)	0.180
Retroversion (°)	50.0 (0.0)	50.0 (0.0)	0.317
ER 0° AB (°)	50.0 (22.5)	50.0 (15.0)	0.317
ER 90° AB (°)	90.0 (0.0)	90.0 (0.0)	0.317
Adduction force (kg)	9.0 (7.3)	10.5 (7.0)	0.039 *

## Data Availability

The data presented in this study are available on request from the corresponding author.
